# Intussusception

**Published:** 1901-03-30

**Authors:** 


### INTUSSUSCEPTION.

Intussusception is a surgical emergency which requires prompt recognition and immediate treatment. Dr. Charles Clubbe,[1] of Sydney, says that twenty-four hours' delay is in such cases actually a matter of life and death, a statement which he illustrates by the fact that of forty-nine cases with which he had had to deal during the past seven years, the average time which elapsed from the onset of the symptoms till the operation averaged twenty-four hours in the successful cases, whereas in the unsuccessful ones the average time was fifty-six hours. In the majority of cases, he says, there may be very little in the appearance of the child when first seen to indicate the serious nature of the condition. What then are the points we must look for? The history is nearly always something to this effect: The baby was quite well, when suddenly it screamed out, turned pale, and soon afterwards vomited. We are often told that the screaming continued only for a short time, that the pallor soon disappeared, but that the child seemed to get frequent attacks of pain. We shall very likely also be told that the child passed a natural motion soon after the first attack of screaming. When first seen by the doctor it has almost certainly been passing some blood by the bowel. This does not generally occur until some hours (from two to ten) after the intussusception has taken place, and it is this passing of blood that alarms the mother. So when a woman brings her baby and says it is passing blood, one must always ask certain questions, and if one gets a history such as the above, one must look out, and must not send the child away with a little physic. Its general condition is apt to mislead. It may lie quite still and play with its toys between the attacks of pain, its pulse rate may be hardly raised, its temperature is probably normal, and it may not look ill. On examination, however, the "sausage-shaped" tumour may be discovered, and if it is not possible to make a satisfactory examination because of the rigidity of the abdominal muscles chloroform must be given. With such a history a child must not be sent away without an examination under an anæsthetic. It is not necessary to examine by the rectum, for in the early stage nothing will be found there, while later one will almost certainly find the "sausage-shaped" tumour in the abdomen. It must be remembered that many a case of intussusception takes place during an attack of diarrhœa, when the history may be only that of a sudden aggravation of the symptoms, that the baby had a screaming fit, and that ever since then it had been passing more blood than before. Many children have no doubt been certified as having died of dysentery or gastro-enteritis when really they have died of unrecognised intussusception. Again, intussusception may exist with hardly any definite symptoms to excite suspicion. In such a case the child may even have passed several normal motions, although, if the intussusception is at all acute after twenty-four hours there will almost always be symptoms of intestinal obstruction.

The treatment lies between injections and operation, the latter taking the form of reduction or resection. Notwithstanding the general disfavour which has of late fallen upon injections, Dr. Clubbe considers them both safe and useful, and he nearly always uses them at whatever stage he sees the case. In the early stage injections have now and then completely reduced the invagination, and there has been no further trouble. This has occurred four times in his forty-nine cases, while he thinks that in all cases an injection properly given reduces the invagination to a certain extent, and thus diminishes the gravity and extent of the subsequent operation.

[1] Brit. Med. Jour., March 23.

